# Effect of Head Rotation on Cerebral Blood Velocity in the Prone Position

**DOI:** 10.1155/2012/647258

**Published:** 2012-09-05

**Authors:** Jakob Højlund, Marie Sandmand, Morten Sonne, Teit Mantoni, Henrik L. Jørgensen, Bo Belhage, Johannes J. van Lieshout, Frank C. Pott

**Affiliations:** ^1^Department of Anaesthesia, Sygehus Nord, 4300 Holbæk, Denmark; ^2^Department of Diagnostic Radiology, Bispebjerg University Hospital, 2400 Copenhagen, Denmark; ^3^Department of Anaesthesia, Bispebjerg Hospital Research Unit for Anaesthesia and Intensive Care (B.R.A.IN), Bispebjerg University Hospital, Bispebjerg Bakke 23, 2400 Copenhagen NV, Denmark; ^4^Department of Clinical Biochemistry, Bispebjerg University Hospital, 2400 Copenhagen, Denmark; ^5^Acute Admissions Unit, Department of Internal Medicine, Laboratory for Clinical Cardiovascular Physiology, AMC Center for Heart Failure, University of Amsterdam, 1105 AZ Amsterdam, The Netherlands; ^6^School of Biomedical Sciences, University of Nottingham Medical School, Queen's Medical Centre, Nottingham NG7 2RD, UK

## Abstract

*Background*. The prone position is applied to facilitate surgery of the back and to improve oxygenation in the respirator-treated patient. In particular, with positive pressure ventilation the prone position reduces venous return to the heart and in turn cardiac output (CO) with consequences for cerebral blood flow. We tested in healthy subjects the hypothesis that rotating the head in the prone position reduces cerebral blood flow. *Methods*. Mean arterial blood pressure (MAP), stroke volume (SV), and CO were determined, together with the middle cerebral artery mean blood velocity (MCA V_mean_) and jugular vein diameters bilaterally in 22 healthy subjects in the prone position with the head centered, respectively, rotated sideways, with and without positive pressure breathing (10 cmH_2_O). *Results*. The prone position reduced SV (by 5.4 ± 1.5%; *P* < 0.05) and CO (by 2.3 ± 1.9 %), and slightly increased MAP (from 78 ± 3 to 80 ± 2 mmHg) as well as bilateral jugular vein diameters, leaving MCA V_mean_ unchanged. Positive pressure breathing in the prone position increased MAP (by 3.6 ± 0.8 mmHg) but further reduced SV and CO (by 9.3 ± 1.3 % and 7.2 ± 2.4 % below baseline) while MCA V_mean_ was maintained. The head-rotated prone position with positive pressure breathing augmented MAP further (87 ± 2 mmHg) but not CO, narrowed both jugular vein diameters, and reduced MCA V_mean_ (by 8.6 ± 3.2 %). *Conclusion*. During positive pressure breathing the prone position with sideways rotated head reduces MCA V_mean_ ~10% in spite of an elevated MAP. Prone positioning with rotated head affects both CBF and cerebrovenous drainage indicating that optimal brain perfusion requires head centering.

## 1. Introduction

Prone positioning of patients during anesthesia is required for a variety of surgical procedures [1]. Furthermore, the prone versus supine position promotes ventilation/perfusion matching in critically ill patients with acute respiratory distress syndrome [2]. When moving a patient into the prone position, arterial pressure usually remains stable whereas a reduction in cardiac output (CO) [3] is attributed to a decrease in venous return [4] by abdominal compression with partial inferior caval vein obstruction [1]. Many anesthetists place the head centered in a headrest during prone positioning especially during more extended procedures, for example, spine surgery. However, since it is less cumbersome and/or the head position may not be considered important many patients are placed with the head positioned to the side. This is especially the case when laryngeal mask airways are used for surgery in the prone position, which is an increasingly used practice [5–9]. In the anesthetized patient, the positioning-related reduction in CO is amplified by positive pressure ventilation. Complications associated with prone positioning often with the head in a sideward-rotated position include occlusion of cervical arteries or veins, and injuries of the cervical spine and peripheral nerves [1, 6, 10]. Particularly, carotid and vertebral artery occlusion and dissection and middle cerebral artery (MCA) infarction have been linked to head rotation or extension [1]. In a recent review, postoperative visual loss as a recognized complication of prone positioning has been linked to hemodynamic alterations [10]. Positive pressure breathing in the supine position reduces cerebral blood flow [11]. Also, prone positioning raises intracranial pressure with potentially adverse consequences for cerebral perfusion and/or cerebral venous drainage [12]. Additional sideward rotation of the head in that position leads to venous compression with potential cerebral venous outflow impairment. In infants, prone positioning with the head rotated versus the supine position with the head centered reduces transcranial Doppler determined MCA blood velocity (MCA *V*_mean_) [13]. Also, rotating of the head increases cerebral blood volume attributed to impairment of venous drainage [14] whereas in adults the effects of head direction during prone positioning have not been studied. We hypothesized that during positive pressure breathing in the prone position, head rotation would reduce MCA *V*_mean_. We set out to investigate systemic hemodynamics and brain arterial and venous characteristics by following MCA *V*_mean_ as an index of cerebral blood flow and jugular vein diameter in the prone position with and without positive pressure breathing and/or head rotation. 

## 2. Materials and Methods

22 healthy volunteers (7 women), aged 24 ± 4 years (mean ± standard deviation), height 182 ± 10 cm, weight 78 ± 12 kg participated in this investigation. All gave their informed consent prior to inclusion in the study. The study was approved by the Ethics Committee for Copenhagen and Frederiksberg (KF 01 287338) and was performed in accordance with the Helsinki Declaration.

MCA *V*_mean_ was measured using a DWL Multiflow 4X Doppler apparatus (DWL, Sipplingen, Germany). The proximal segment of the right middle cerebral artery was insonated at a depth of 45–57 mm through the “postero-temporal window.” After the optimal signal-to-noise ratio was established the probe was fixed to the head with adhesive ultrasonic gel (Tensive, Parker Laboratories Inc., Fairfield, NJ, USA) and firmly secured using a custom-made headband. A clear, soft, plastic mask (VBM Medizintechnik GmbH, Sulz, Germany) was fitted over each subject's nose and mouth using elastic bands around the back of the head: positive pressure breathing was applied using a Whisperflow fixed flow generator using pressurized room air and Whisperflow isobaric continuous positive airway pressure (CPAP) valves with 10 cmH_2_O opening pressure (Caradyne, Galway, Ireland). 

Finger arterial pressure was measured by a Finometer apparatus (Finapres Medical Systems, Amsterdam, The Netherlands). The cuff was applied to the midphalanx of the middle finger of the dominant hand and placed at heart level. 10 subjects had a 1.1 mm inner diameter arterial catheter placed in the radial artery of the nondominant hand. Blood gases were analyzed on an ABL 800-series apparatus (Radiometer, Copenhagen, Denmark). All variables were A/D converted, sampled at a rate of 100 Hz, 16 bit (PCI-Base 1000 hardware and NextView software (BMC Messsysteme GmbH, Berlin, Germany)) by PC and stored for off-line analysis.

The cross-sectional area of both internal jugular veins (*A*_jug_) were measured using a LOGIQ 500 pro series ultrasound machine (GE Medical Systems, Milwaukee, WI, USA) adapting the Cirovic method for measuring jugular cross-sectional area [15]. All measurements were performed with a 9 MHz linear probe perpendicular to the skin surface at skin markings made equidistantly between the mastoid process and the jugular notch. Compression of the vein was avoided by applying abundant ultrasonic gel thus minimizing skin pressure by the probe. The ultrasonic picture was frozen at end-expiration for off-line analysis.

### 2.1. Protocol

Following instrumentation, the subject was placed supine on an Alphamaquet 1150 operating table (Maquet, Rastatt, Germany) and rested for 30 minutes. Measurements were performed in 3 body positions in random order with and without supplementation of 10 cmH_2_O CPAP: (1) supine, (2) prone with the head centered, supported by the brow and cheekbones in a horseshoe headrest with supplemental padding, and (3) prone with the head rotated ~80 degrees to the right resting it on a soft pillow. Each measurement cycle consisted of 4 min of ambient pressure breathing and 4 minutes of positive pressure breathing; also in random order. Baseline was the supine position with 0 cmH_2_O of CPAP. Arterial blood gas samples were obtained during the last 15 seconds of each intervention. Within the last 2 minutes of each intervention, the cross-sectional areas of both internal jugular veins (*A*_jug_) were quantified. 

### 2.2. Data Analysis

The finger arterial pressure curve was analyzed using Beatscope software (Finapres Medical Systems, Amsterdam, The Netherlands). Beat-to-beat systolic, diastolic, and mean arterial pressures (MAP), as well as stroke volume (SV), were computed from the arterial pressure pulse wave by off-line Model flow analysis [16]. This method computes an aortic flow waveform by simulating a nonlinear, time-varying model of the aortic input impedance, thereby calculating SV. Changes in SV and CO are tracked accurately, whereas absolute values require calibration against a Fick principle method [17–19]. MAP was obtained as the integral of pressure over one beat divided by the corresponding beat length. Heart rate (HR) was the reciprocal of the interbeat interval. CO was the product of SV and HR. All variables were transformed to equidistantly resampled data at 1 Hz by polynomial interpolation and averaged over 30 second intervals just prior to the end of each intervention. Changes are reported relative to baseline.

### 2.3. Statistical Analysis

Data obtained as averages over a sampling period are expressed as mean ± SEM. Data from a single sampling point (jugular luminal area) are expressed as mean ± SD. The effect of positive pressure breathing and head position on cerebral and systemic outcome variables was tested using paired *t*-test between interventions of interest (prone versus supine; prone with the head turned versus prone; ambient versus positive pressure breathing in the different positions). A *P*-value < 0.05 was considered to indicate a statistically significant difference.

## 3. Results

### 3.1. Prone Position and Head Rotation

**Figure 1:**
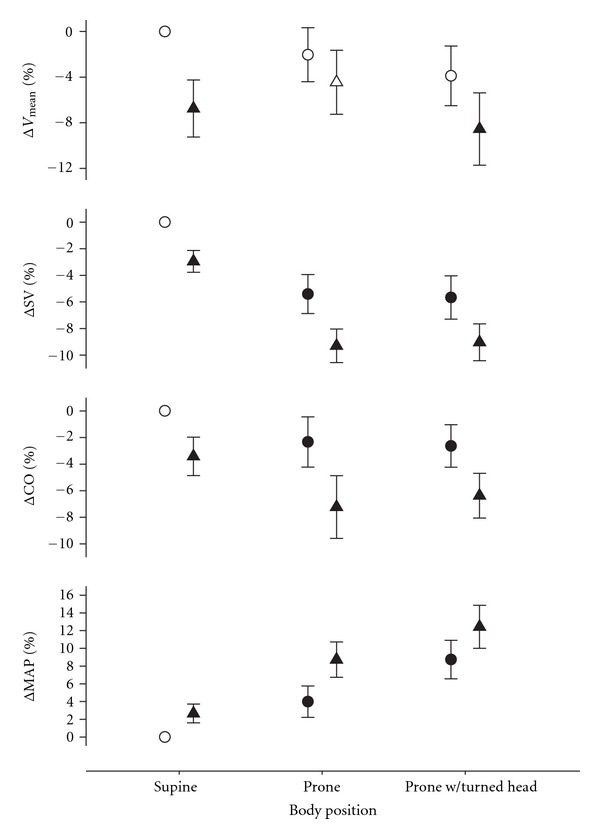
Systemic and cerebral circulatory responses to continuous positive airway pressure (CPAP) at different body positions. Changes in middle cerebral artery mean blood velocity (ΔMCA *V*_mean_), mean arterial pressure (ΔMAP), cardiac stroke volume (ΔSV), and cardiac output (ΔCO) during CPAP 0 cmH_2_O (circles), and 10 cmH_2_O (triangles). A filled circle indicates a statistically significant difference from supine. A filled triangle indicates a statistically significant effect of 10 cmH_2_O CPAP.

SV and CO decreased from supine to prone position together with an increase in MAP. MCA *V*_mean_, PaCO_2_ and PaO_2_ remained unchanged (Table 1, Figure 1). Head rotation to the right side in the prone position increased MAP further but left SV, CO, MCA *V*_mean_, PaCO_2_, and PaO_2_ unchanged.

### 3.2. Positive Pressure Breathing

In the supine position, addition of 10 cmH_2_O of CPAP increased MAP slightly and more so when prone. SV and CO decreased with positive pressure breathing regardless of body position. MCA *V*_mean_ decreased with positive pressure breathing in the supine position. CPAP increased PaO_2_ mainly in the supine position, while PaCO_2_ remained unchanged (Table 1, Figure 1).

### 3.3. Positive Pressure Breathing and Head Rotation

The reductions in SV and CO were similar to those seen in the prone position with the head centered, while MAP reached the highest values. MCA *V*_mean_ reached a nadir with positive pressure breathing in the head-rotated prone position (Table 1, Figure 1). PaCO_2_ remained unchanged.

### 3.4. Jugular Vein Diameters

In the supine position and in the prone position with the head centered, jugular vein diameter was ~40–75% larger on the right side. Prone positioning increased jugular vein diameters on both sides but decreased with rightward head rotation especially on the right side (Table 1).

## 4. Discussion

The results of this study provide insight into the effects of prone positioning, head rotation and positive pressure breathing on brain perfusion and brain venous drainage. The prone position with sideways rotated head and positive breathing narrowed the ipsilateral internal jugular vein together with a ~10% reduction in MCA *V*_mean_ in spite of an elevated MAP. These results suggest that for this commonly applied anesthetic approach both cerebral blood flow and cerebrovenous drainage are optimal only with the head centered. 

Transcranial Doppler ultrasound monitors blood peak flow velocity rather than volume flow, and changes in the diameter of the insonated vessel could modulate velocity independently of volume flow. During craniotomy, the diameter of the MCA remains unchanged by even large changes in arterial pressure [20]. Constancy of the diameter of the MCA as determined with magnetic resonance imaging during changes in carbon dioxide tension and in simulated orthostasis further supports that the MCA is not involved in regulation of cerebral vascular resistance [21] linking changes in MCA *V*_mean_ to those in cerebral blood flow [22].

All Finometer and TCD variables were A/D converted and stored for off-line analysis. The off-line analysis involved detection and manually removal of artifacts with subsequent semiautomated data extraction. This semiautomated process leaves little potential for introduction of bias. Jugular vein areas were obtained off-line by manually tracking the outline of the vein. Potential for bias exist as we consider blinding impractical because the position of the head was revealed by the distortion of the structures in the ultrasonographic picture.

 With prone position, we observed a small reduction in cardiac stroke volume and output (Figure 1) in accordance with an earlier report in anesthetized patients likely reflecting reduced venous return [23]. The approximate doubling of internal jugular vein cross-sectional area when positioned below heart level suggests passive gravitational jugular vein dilation (Table 1). However, MCA *V*_mean_ was largely unaffected (Figure 1).

Head rotation in the prone position increased MAP by ~4 mmHg (Figure 1) possibly reflecting unloading of carotid baroreceptors by altered pressure from the soft tissues with a reflex increase in sympathetic nervous activity [24]. Head-down rotation engages the otolith organs and vestibular otolith stimulation may increase sympathetic activity during baroreflex unloading [25]. Positive pressure breathing also enhances resting muscle sympathetic activity and the reduction of urinary output and sodium excretion associated with prolonged positive pressure breathing has been attributed to cardiopulmonary receptor unloading [26]. A contribution of enhanced sympathetic activity modulating cerebral vascular tone may be considered. The increase in MAP from supine to the prone position presumably by increased sympathetic activity was not accompanied by changes in MCA *V*_mean_ reflecting integrity of cerebrovascular autoregulation mechanisms. In contrast, positive pressure breathing reduced cardiac output together with MCA *V*_mean_ whereas MAP increased. A restricted CO that challenges MAP limits flow to the brain [27, 28], but under conditions where MAP is not challenged by a restricted CO, the influence of sympathetic stimulation on CBF is not manifested [29]. Thus, in this study, we consider an altered cerebral venous outflow resistance rather than sympathetic activity to dominate CBF.

With the head-turned constancy of MCA *V*_mean_ was maintained notwithstanding a ~50% reduction in *A*_jugR_. The cerebral venous system is characterized by many collaterals and void of valves directing blood flow [30], explaining why compression of one jugular vein was of no consequence for cerebral blood flow as long as the central blood volume was maintained. Rotation per se appears to be of little influence for the cerebral blood supply since rotation of the head in the supine position leaves the diameter of the common and internal carotid artery unchanged [31]. Our subjects had their head supported in a horseshoe headrest avoiding external pressure on the neck, and we consider compromised arterial cerebral inflow unlikely to have caused the reduction in MCA *V*_mean_. However, carotid compression cannot be excluded in prone-positioned anesthetized patients, for example, by external compression of supporting pillows and/or inappropriate extention/rotation. Only ~40% of normal subjects have a complete Circle of Willis maintaining blood flow to the contralateral side of the brain with unilateral occlusion [32]. Thus, variations in the Circle of Willis may have influenced cerebral hemodynamics following head rotation in the prone position. Furthermore, especially with incomplete Circle of Willis, the side of head rotation may be of importance for the hemodynamic response, but this was not investigated in our study.

During positive pressure breathing when supine MCA *V*_mean_ was reduced together with SV and CO conforming an earlier report [11] and reflecting a reduction of the central blood volume imposed by positive pressure breathing [33]. Under the conditions of this study, absence of changes in PaCO_2_ with CPAP renders an effect of CO_2_ on CBF unlikely. 

During positive pressure breathing in the head centered prone position MCA *V*_mean_ was maintained despite a further reduction in SV and CO. This observation is compatible with the notion that an increase in cerebral perfusion pressure in the prone position may sustain CBF (Figure 1) [34, 35]. 

MCA *V*_mean_ reached a nadir of ~10% below baseline in the head-rotated prone position with positive pressure breathing. This reduction is within the range observed under everyday physiological challenges, that is, standing up [36]. By comparison a ~50% decline is associated with clinical ischemia [22] and syncope [33]. As general anesthesia has a favorable supply/demand profile for cerebral oxygen flux—even during induction, where the cardiovascular depression often is largest [37], it could be argued that the changes induced by prone position are of little clinical relevance to most patients. However, even small reductions in cerebral blood supply could be deleterious in the setting of an already compromised cerebral perfusion, for example, in elderly subjects with vascular disease [12, 34, 35, 38]. 

The applied pressure of 10 cmH_2_O of CPAP is (on average) similar to the prevailing intrathoracic pressure when ventilating healthy patients in the prone position [39] whereas in the presence of lung disease or marked obesity inflation pressures may surpass this value. The reduction of the central blood volume as an accompanying effect of positive pressure breathing did reduce CO and MCA *V*_mean_. These new findings are of clinical relevance since they indicate that CBF may be compromised by venous compression in the head-turned prone position when the central blood volume is challenged by positive pressure breathing.

In healthy awake individuals, the prone position with positive pressure breathing reduced MCA *V*_mean_ especially when the head was rotated to the side. These results may have implications for the anesthetized and ventilated patient. The hypothesis that in the prone position both CBF and cerebrovenous drainage are optimal only with the head centered should be tested in patients undergoing general anesthesia. 

## Figures and Tables

**Table 1:** Systemic and cerebral circulatory and jugular venous responses to continuous positive airway pressure (CPAP) at different body positions.

Position	Supine		Prone		Prone w/head turned	
CPAP	0 cmH_2_O	10 cmH_2_O	0 cmH_2_O	10 cmH_2_O	0 cmH_2_O	10 cmH_2_O
ΔMCA *V*_mean_ (%)	0 ± 0	−6.8 ± 2.5^†^	−2.0 ± 2.4	−4.4 ± 2.8	−3.9 ± 2.6	−8.6 ± 3.2^†^
ΔSV (%)	0 ± 0	−3.0 ± 0.8^†^	−5.4 ± 1.5*	−9.3 ± 1.3^†^	−5.7 ± 1.6*	−9.0 ± 1.4^†^
ΔCO (%)	0 ± 0	−3.4 ± 1.4^†^	−2.3 ± 1.9*	−7.2 ± 2.4^†^	−2.6 ± 1.6*	−6.4 ± 1.7^†^
HR (bpm)	59 ± 2	59 ± 2	61 ± 2	61 ± 2	62 ± 2	61 ± 2
MAP (mmHg)	78 ± 2	79 ± 2^†^	80 ± 3*	84 ± 3^†^	84 ± 3*	87 ± 2^†^
*A*_ jugR_(cm^2^)	1.0 ± 0.5	1.4 ± 0.5^†^	1.9* ± 0.8	2.1 ± 0.9^†^	0.9* ± 1.1	1.2 ± 1.0^†^
*A*_ jugL_ (cm^2^)	0.7 ± 0.4	0.9 ± 0.5^†^	1.3* ± 0.7	1.2 ± 0.6^†^	1.1* ± 0.6	1.2 ± 0.6^†^

Changes in middle cerebral artery mean blood velocity (ΔMCA *V*_mean_), mean arterial pressure (MAP), cardiac stroke volume (ΔSV), cardiac output (ΔCO), heart rate (HR), and right and left internal jugular vein cross-sectional area (*A*_jugR_, *A*_jugL_) during CPAP 0 cmH_2_O and 10 cmH_2_O. *Significant different from supine, *P* < 0.05. ^†^Significant different from 0 cmH_2_O CPAP.
